# Impact of lead exposure on global chronic kidney disease attributable to hypertension: deaths and disability-adjusted life years from 1990 to 2021 and projected trends for 2022–2036

**DOI:** 10.3389/fpubh.2025.1635877

**Published:** 2025-09-23

**Authors:** Yujun He, Yaling Zheng, Weiwei Tang, Bowen Xing, Hui Xu, Jiujie He, Wei Mai, Xiaoyi Wang, Miao Zhou

**Affiliations:** ^1^Department of Traditional Chinese Medicine, Taizhou Hospital of Zhejiang Province Affiliated to Wenzhou Medical University, Taizhou, Zhejiang, China; ^2^Preventive Health Center, The First People's Hospital of Chenzhou City, Hunan, China; ^3^Faculty of Acupuncture, Moxibustion and Tui Na, Guangxi University of Chinese Medicine, Nanning, Guangxi, China; ^4^Department of Traditional Chinese Medicine, Guangxi Medical University Cancer Hospital, Nanning, China

**Keywords:** lead exposure, CKD due to hypertension, global burden of disease, predictive analysis, socio-demographic index

## Abstract

**Background:**

Chronic kidney disease (CKD) due to hypertension represents a major global health challenge, with lead exposure exacerbating this burden.

**Objective:**

This study aims to analyze the global burden of lead-attributable hypertensive CKD from 1990 to 2021 and project trends over the next 15 years.

**Method:**

Data from the Global Burden of Disease (GBD) 2021 study were utilized, focusing on mortality and disability-adjusted life years (DALYs). Trends were analyzed across 204 countries/territories, categorized into 21 GBD regions and five socio-demographic index (SDI) quintiles. Detailed analyses of case numbers, age-standardized rates (ASRs), age groups, sex differences, and temporal trends were conducted. Pearson correlation analysis was applied to assess the association between SDI and disease burden, frontier analysis was used to evaluate country-specific burden levels, and autoregressive integrated moving average (ARIMA) models were employed to project future trends (2021–2036).

**Results:**

From 1990 to 2021, global deaths from lead-attributable hypertensive CKD increased from 16,932 to 52,839 (a 212.07% rise), with age-standardized mortality rate increasing from 0.481 to 0.641 per 100,000 (EAPC = 1.05). DALYs rose from nearly 470,000 to 1.17 million (a 150.98% increase), with age-standardized DALYs rate increasing from 11.825 to 13.725 per 100,000 (EAPC = 0.55), with marked regional disparities. Older adults and males bore a heavier burden. The highest burdens were observed in low SDI regions, while high SDI regions had the lowest burdens, with a minor inflection point at an SDI of approximately 0.5. Frontier analysis revealed substantial heterogeneity in disease burden across countries. Projection analyses indicated a potential reduction in disease burden over the next 15 years.

**Conclusions:**

Addressing lead exposure is critical to mitigating the burden of CKD due to hypertension. Targeted interventions tailored to SDI levels and lessons from frontier-line countries are recommended to achieve equitable burden reduction.

## 1 Introduction

Chronic kidney disease (CKD) has emerged as a major global health issue, imposing a substantial burden on healthcare systems and public health worldwide. Hypertensive nephropathy, a long-term complication of poorly controlled hypertension (1, 2), represents the second leading cause of CKD after diabetic nephropathy (3, 4) and serves as a critical contributor to the deterioration of renal function (5). According to the Global Burden of Disease Study (GBD), in 2021, there were approximately 674 million prevalent CKD cases globally, with an age-standardized prevalence of approximately 8,000 cases per 100,000 person, underscoring its significant mortality and morbidity burden (3). However, the complex interplay between hypertension and CKD poses a profound global challenge for preventing hypertension-related CKD progression.

Lead, a ubiquitous environmental contaminant, has a long history of human exposure due to its extensive use in industries such as mining, manufacturing, and gasoline production (6). As a toxic heavy metal, lead acts as an independent risk factor for the progression of hypertension through mechanisms such as activation of the renin-angiotensin system and oxidative stress-induced damage to vascular endothelium (7). Meanwhile, lead also exerts nephrotoxicity through multiple mechanisms such as oxidative stress (8). Lead poses risks to both hypertension and kidney damage, so in patients with hypertensive nephropathy, lead exposure exacerbates the burden of the disease. A recent analysis of 2019 data revealed a substantial disease burden of lead exposure on CKD, with notable regional disparities (9). In recent years, increasing attention has been paid to the damage of lead exposure to the circulatory system. A large-sample study based on U.S. NHANES data found a significant non-linear association between blood lead levels and subclinical myocardial injury, especially when blood lead levels exceed 3.8 μg/dl, with a markedly increased risk of myocardial injury. This finding suggests that lead may indirectly promote hypertension and its renal complications by inducing cardiovascular dysfunction, providing important epidemiological evidence for exploring the “lead exposure—hypertension—CKD” pathway (10). However, the specific impact of lead on the global burden of hypertension-attributed CKD remains incompletely elucidated and requires updated data to inform contemporary public health strategies.

From 1990 to 2021, significant changes have occurred in global lead exposure patterns, public health policies, and CKD epidemiology. This period has witnessed both notable advancements, such as the phased elimination of leaded gasoline in many countries and improvements in occupational safety regulations, and persistent challenges, including ongoing lead contamination in certain regions and populations (11). Understanding how these dynamic factors have shaped the relationship between lead exposure and hypertension-induced CKD globally is critical to informing targeted prevention and intervention strategies. This study aims to systematically analyze the impact of lead exposure on the global burden of hypertension-induced CKD from 1990 to 2021 and project trends in the global burden for the next 15 years. The findings have the potential to enhance our understanding of the environmental determinants of hypertension-attributed CKD, guide policy-making for lead exposure reduction, and ultimately contribute to global efforts to ameliorate renal health and mitigate the burden of hypertension-related CKD.

## 2 Material and methods

### 2.1 Research population and data compilation

The GBD 2021 study adopts a comprehensive approach to estimate disease burden, including integrating multi-source data, using the DisMod-MR model for disease modeling, and applying Bayesian statistical frameworks and Monte Carlo methods to quantify uncertainties. These methods collectively form a systematic analytical framework capable of generating comparable and consistent estimates of disease burden (12). The GBD 2021 database classifies CKD according to the International Classification of Diseases, 10th Revision (ICD-10). The ICD-10 codes relevant to CKD include D63.1, E10.2, E11.2, E12.2, E13.2, E14.2, I12-I13.9, N02-N08.8, N15.0, N18-N18.9, and Q61-Q62.8. Among them, specifically referring to renal damage directly caused by hypertension, which falls within the category of hypertension-induced CKD (13). Our study focuses on two key epidemiological disease burdens related to hypertension-induced CKD attributable to lead exposure: mortality and Disability-Adjusted Life Years (DALYs). The analysis included 204 countries and territories, grouped into 21 GBD regions based on geographic proximity and subsequently classified into five categories according to the socio-demographic index (SDI).

### 2.2 Data analysis

#### 2.2.1 Overview

We initiated our data analysis by assessing the dataset's structure, estimating numbers and rates for essential metrics including deaths and DALYs of lead-attributable hypertensive CKD at global, regional, and national levels. We subsequently analyzed the measure variations from 1990 to 2021 across multiple regions. This was executed for both case numbers and age-standardized rates (ASRs) (ln[ASR] = a + bx + ε, where x is the calendar year) per 100,000. To determine relative changes from 1990 to 2021, we utilized the formula: Relative change (%) = [(Value in 2021 – Value in 1990)/Value in 1990] ^*^ 100%. This was executed for both case numbers and ASRs per 100,000 individuals (14).

The Estimated Annual Percentage Change (EAPC) was utilized to quantify trends in age-standardized rates (ASRs), which was derived via a generalized linear model under the assumption of a Gaussian distribution (15). For EAPC calculation, calendar year was designated as the explanatory variable X, while the natural logarithm of age-standardized rates (ln[ASR]) functioned as the dependent variable Y to fit the data to the regression equation y = a + bx + ε. Subsequently, EAPC was computed using the parameter β from the fitted regression line through the formula: EAPC = 100 × [exp(β) – 1] (16, 17). This computational approach is valid only when ASR changes remain consistent throughout the entire observation period. Statistical hypothesis testing was performed to assess the computed EAPC and account for the impact of random variability. The hypothesis testing for EAPC is equivalent to that for the slope of the fitted regression line; specifically, EAPC is deemed valid when the slope exhibits statistical significance. The hypothesis testing process for EAPC involved a *t*-test on the slope b of the fitted line, expressed as tb = b/sb (where b represents the slope of the line and sb denotes the standard error of slope b), with the degrees of freedom V being equal to the number of calendar years minus 2. Given that the standard error of slope b influences both the slope of the fitted line and EAPC, the 95% confidence intervals (CIs) for EAPC were calculated in accordance with the methodologies described in references (18, 19).

#### 2.2.2 Relationship between lead-attributable hypertensive CKD burden and SDI

The SDI is a composite indicator developed by GBD researchers to assess a region's socio-economic status. It integrates per capita income, educational achievement, and fertility rates into a unified statistic ranging from 0 to 1, signifying the socio-economic vitality and progress of a region or nation (14). Increased SDI levels signify enhanced socio-economic conditions and health outcomes. The SDI categorizes regions into quintiles: low (0-0.454743), low-middle (0.454743-0.607679), middle (0.607679-0.689504), high-middle (0.689504-0.805129), and high (0.805129-1). The correlation between the burden of lead-attributable hypertensive CKD and the SDI will be analyzed using Pearson correlation analysis (20).

#### 2.2.3 Frontier analysis

To minimize redundancy while preserving technical precision, we applied stochastic frontier analysis to quantify the gap between observed and theoretically attainable disease burdens. A benchmark frontier was constructed from countries exhibiting the minimum burden at each level of the SDI. This method departs from conventional inequality metrics by isolating nations whose disease burdens deviate upward from the frontier within homogeneous SDI strata, thereby signaling potential systemic failures in health-care delivery or sub-optimal risk-factor control. Such framing explicitly encourages policy learning among SDI-comparable peers and disentangles macroeconomic development from intrinsic capacity for disease containment, thus generating actionable evidence for resource allocation that conventional cross-national comparisons cannot provide. These leading regions then served as standards and goals for others. For every country and territory, we calculated the “effective difference.” This value represented the disparity between the existing lead-attributable hypertensive CKD burden and the potential one, with adjustments made according to the SDI (21). The calculation method for cutting-edge analysis is based on Guan et al.'s method (22). In this section, the R software package (version 4.2.3) and JD_GBDR (V2.22, Jingding Medical Technology Co., Ltd.) was used for the drawing of the figures.

#### 2.2.4 Predictive analysis

To forecast future trends in the burden of lead-attributable hypertensive CKD, we employed the autoregressive integrated moving average (ARIMA) model. This model leverages the autocorrelation of time-series data to predict future values based on historical observations. The core principle of ARIMA is that data series are time-dependent random variables, characterized by their autocorrelation structure. For the model to be effective, the time series must be stationary and stochastic, with a mean of zero (23, 24). Predictive analyses were conducted using R version 4.3.3.

## 3 Results

### 3.1 Overview of the global burden

#### 3.1.1 Results of the global and regional trend analysis for lead-attributable hypertensive CKD

From 1990 to 2021, the global burden of lead-attributable hypertensive CKD showed a significant upward trend in both mortality and DALYs. Specifically, the number of global deaths increased from approximately 17,000 to 53,000, representing a 212.07% increase, with the age-standardized mortality rasterizing from 0.481 to 0.641 per 100,000 population (EAPC = 1.05). Concurrently, DALYs increased from approximately 470,000 to 1.17 million, a 150.98% increase, with the age-standardized DALYs rate increasing from 11.825 to 13.725 per 100,000 population (EAPC = 0.55) (Supplementary Tables S1, S2).

When stratified by the SDI, marked disparities in disease burden were observed. Low SDI regions consistently bore the heaviest ASR burden, with a mortality ASR of 1.622 per 100,000 and a DALYs ASR of 32.009 per 100,000 in 2021, whereas high SDI regions exhibited the lowest ASRs (mortality ASR: 0.271 per 100,000; DALYs ASR: 5.101 per 100,000). In 2021, middle SDI regions had the highest number of deaths (19110) and DALYs (422325). High SDI regions showed the largest increases in both deaths (267.36%) and DALYs (176.49%), while low SDI regions had the smallest increments (Supplementary Tables S1, S2).

At the regional level, South Asia had the highest number of deaths (9166) and DALYs (258616) in 2021, while North Africa and the Middle East exhibited the highest ASRs for mortality (2.134 per 100,000) and DALYs (38.352 per 100,000). Oceania reported the lowest number of deaths (5) and DALYs (2.340), with Eastern Europe having the lowest ASRs for mortality (0.034 per 100,000) and DALYs (1.186 per 100,000). In terms of trends, Central Asia showed the fastest growth in mortality ASR (EAPC = 3.40), and high-income North America had the fastest growth in DALYs (EAPC = 2.13), whereas Eastern Sub-Saharan Africa exhibited negative growth in both metrics (Supplementary Tables S1, S2).

Regarding sex differences, males consistently had higher numbers of deaths, DALYs, and corresponding ASRs than females in both 1990 and 2021. However, females showed greater increases in burden, with a 235.87% rise in deaths and a 167.39% increase in DALYs, compared to 196.78% and 141.03% for males, respectively (Supplementary Tables S1, S2).

**Figure 1:**
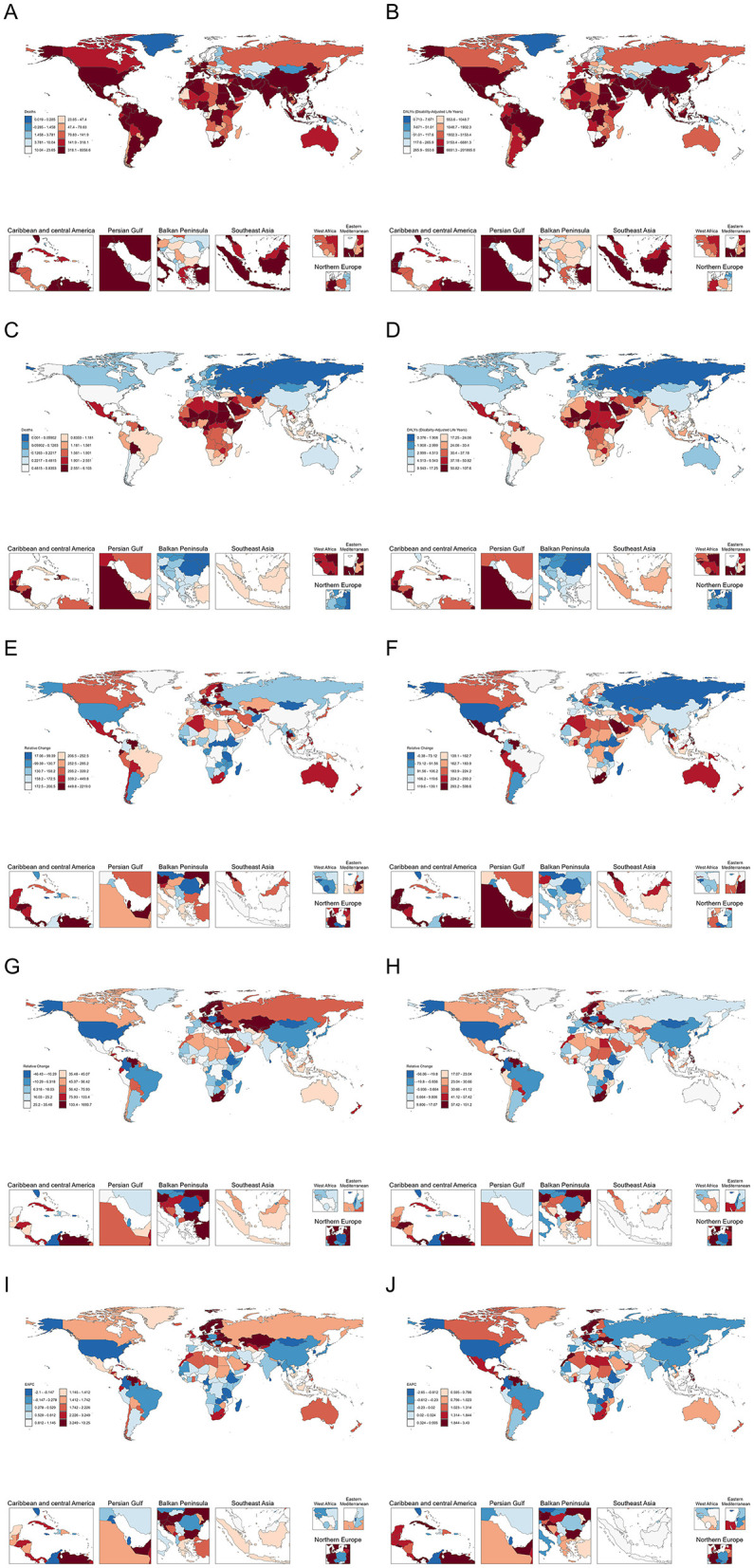
The number of deaths **(A)** and DALYs **(B)** lead-attributable hypertensive CKD in 2021; The ASR of deaths **(C)**, and DALYs **(D)** lead-attributable hypertensive CKD in 2021; The relative change of number of deaths **(E)** and DALYs **(F)** lead-attributable hypertensive CKD from 1990 to 2021; The relative change of ASR of deaths **(G)** and DALYs **(H)** lead-attributable hypertensive CKD from 1990 to 2021; The EAPC of deaths **(I)** and DALYs **(J)** of lead-attributable hypertensive CKD from 1990 to 2021.

The numbers of deaths and DALYs, along with ASR, for lead-attributable hypertensive CKD in 2021, and EAPC, from 1990 to 2021 across 204 countries and territories globally are shown in Figure 1.

#### 3.1.2 Results of the trends by year, sex, and age for lead-attributable hypertensive CKD

**Figure 2:**
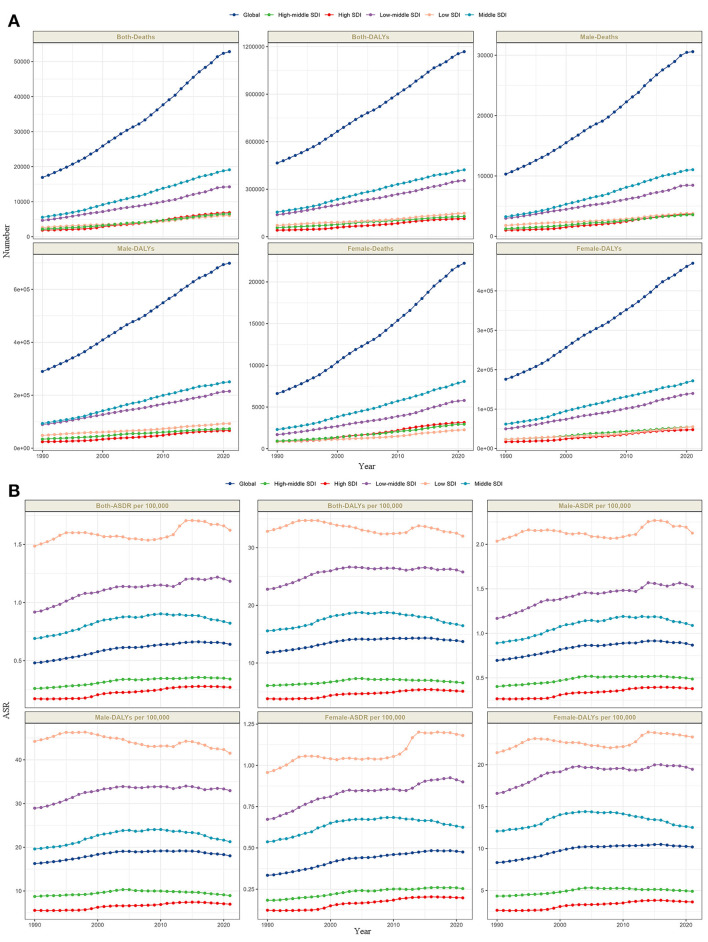
The trends of number in deaths and DALYs **(A)**, and ASR in deaths and DALYs **(B)** lead-attributable hypertensive CKD categorized by global and 5 SDI regions from 1990 to 2021.

From 1990 to 2021, the absolute numbers of deaths and DALYs from lead-attributable hypertensive CKD have increased annually globally and across all five SDI regions (Figure 2A). The ASR also showed an overall slow upward trend. Notably, a subtle gender disparity emerged in Low SDI regions: the age-standardized DALYs rate (ASDR) trended downward in males but upward in females, with the overall trend exhibiting multiple fluctuations (Figure 2B).

**Figure 3:**
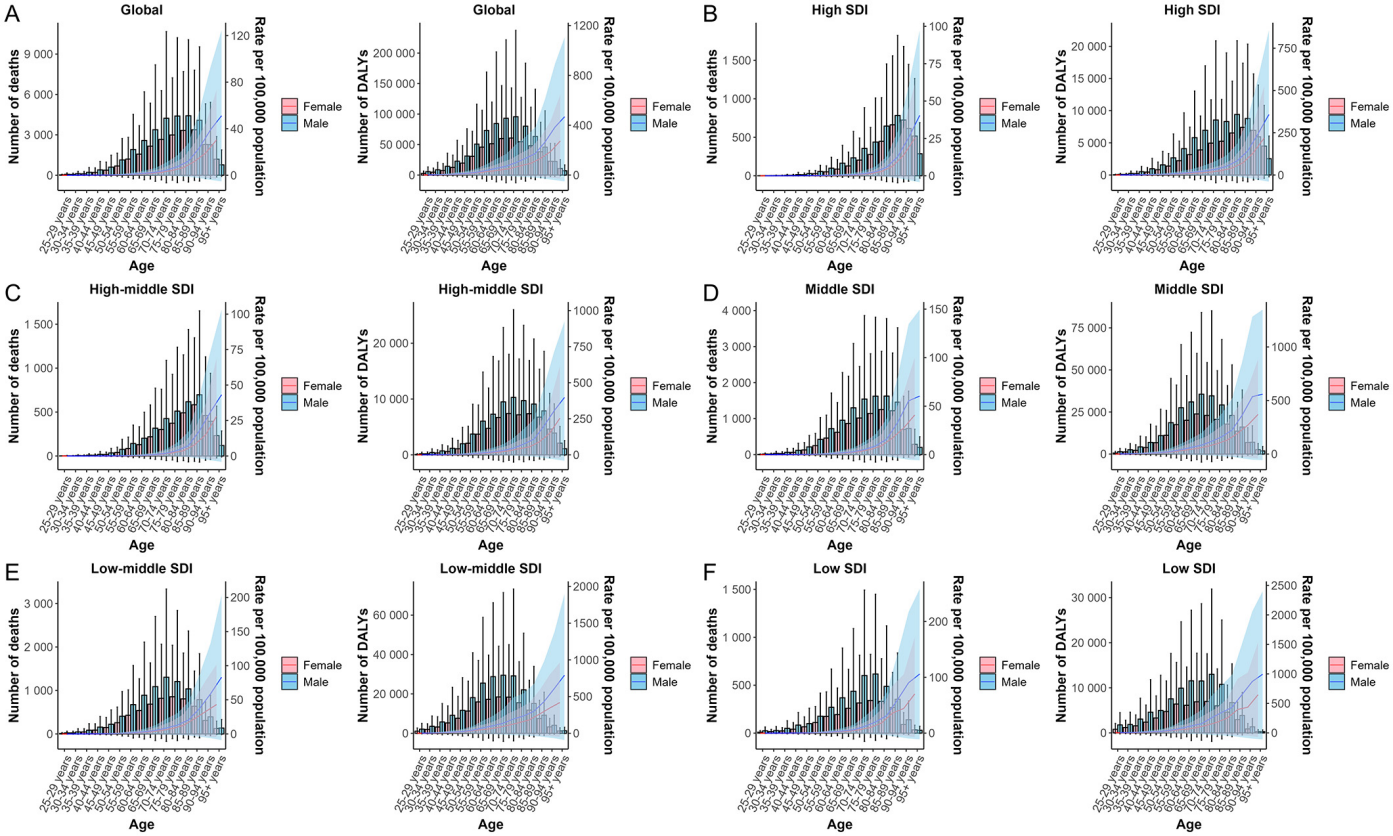
The trends of case number and ASR in deaths and DALYs for lead-attributable hypertensive CKD across different genders, female and male, by age groups in global and 5 SDI regions. Global **(A)**, high SDI **(B)**, high-middle SDI **(C)**, middle SDI **(D)**, low-middle SDI **(E)**, and low SDI **(F)**.

Globally, analyses of lead-attributable hypertensive CKD across different age groups and sexes revealed characteristic trends. For mortality, the number of deaths was higher in males than females before the 85–89 age group, with nearly equal rates between sexes at 85–89 years, after which female deaths exceeded males. For DALYs, this pattern extended to the next age bracket: males had higher DALYs than females before 90–94 years, with parity at 90–94 years, followed by higher female DALYs. ASR for both mortality and DALYs increased with age across all groups, with consistently higher values in males than females (Figure 3A). The five SDI regions exhibited trends comparable to the global pattern (Figures 3B–F).

### 3.2 Results of the relationship between lead-attributable hypertensive CKD burden and SDI

**Figure 4:**
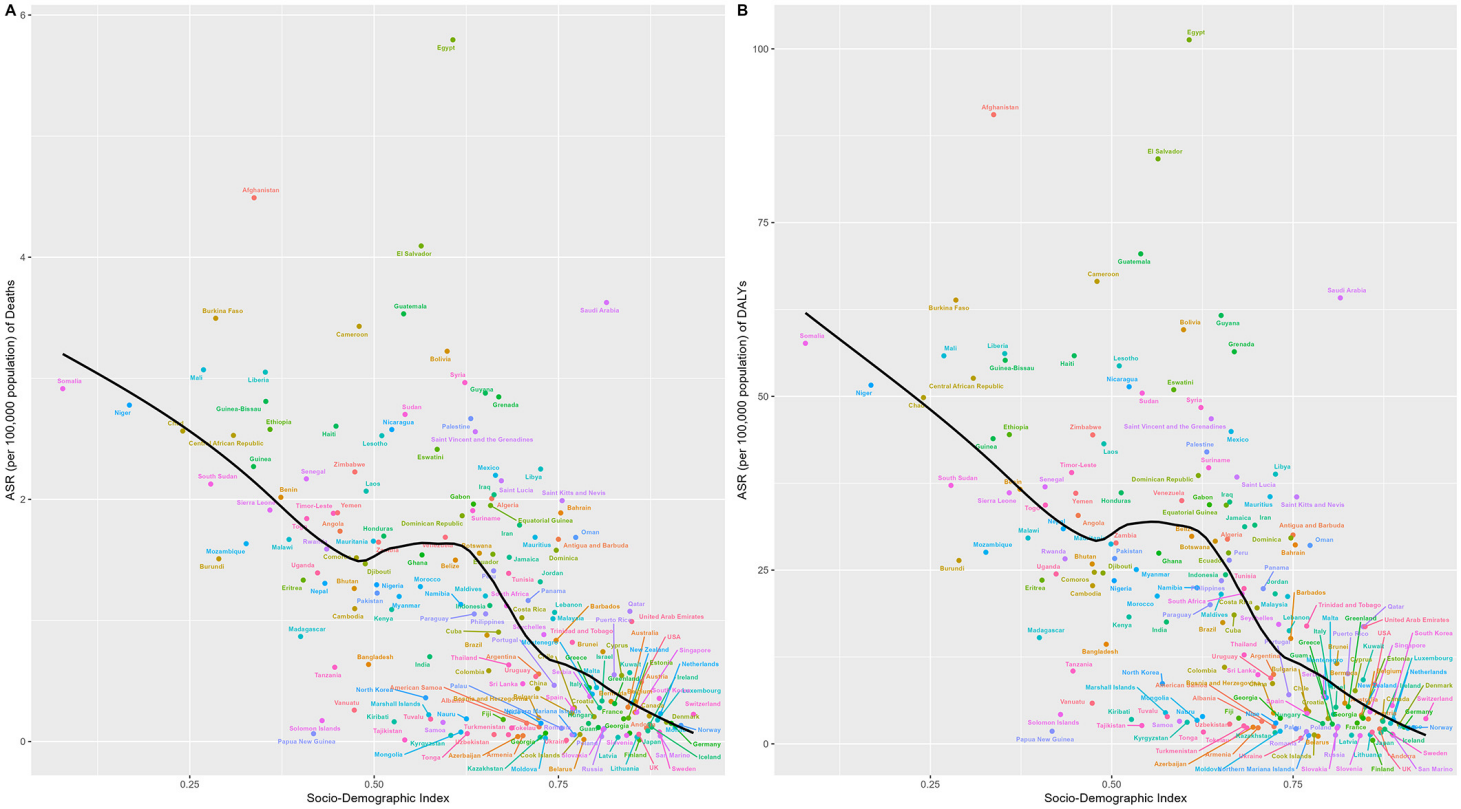
Relationship between SDI and the burden of lead-attributable hypertensive CKD: results of deaths **(A)** and DALYs **(B)** across 204 countries and territories. The black line represents the fitted curve, and each dot represents a country or region.

Globally, the SDI exhibited a significant association with ASR of mortality and DALYs for hypertension-induced CKD attributable to lead exposure, with mortality and DALYs showing nearly identical trends. Notably, both mortality and DALYs burdens were heaviest in low SDI regions and significantly lighter in high SDI regions (both *P* < 0.0001). However, a minor inflection point emerged at an SDI of approximately 0.5, after which the burden increased with rising SDI between 0.5 and 0.6 until reaching an SDI of around 0.6, where it reversed to a downward trend with further increases in SDI (Figure 4).

### 3.3 Results of the frontier analysis for lead-attributable hypertensive CKD

**Figure 5:**
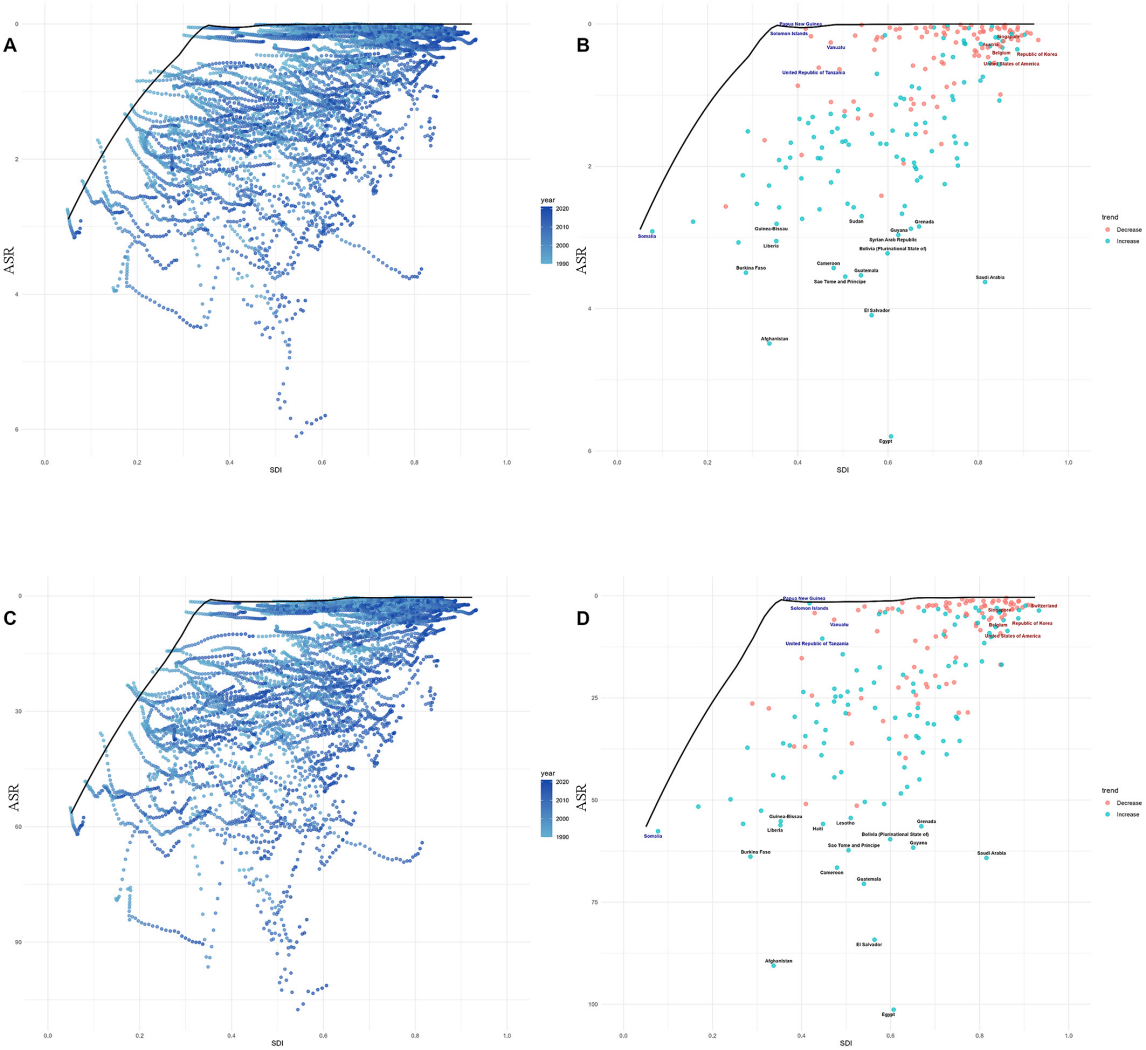
Frontier analysis, represented by the solid black line, examines the relationship between the SDI, the age—standardized rate (ASR), and deaths **(A, B)** as well as DALYs **(C, D)** in the context of lead—attributable hypertensive CKD. The color gradients in **(A, C)** show the change in years, with light colors representing 1990 and the darkest colors representing 2021. In **(B, D)**, each dot represents a specific country or region in 2021. The top 15 countries with the largest deviations from the frontier are labeled in black text. Countries with a low SDI (>0.455) and the smallest deviations from the frontier are highlighted in blue text, while countries with a high SDI (>0.805) and significant deviations in terms of development level are highlighted in red text. The direction of change in the ASR from 1990 to 2021 is indicated by the color of the dots: red dots represent a decrease, whereas blue dots represent an increase.

Frontier analysis revealed the impact of the SDI on ASR of hypertension-induced CKD attributable to lead exposure: for mortality, ASR in low-middle SDI regions generally diverged further from the frontier line with increasing years, while in high-middle SDI regions, countries/territories at the frontier showed little temporal deviation from the frontier line, whereas those already distant from the frontier tended to drift further away over time (Figure 5A). DALYs exhibited an identical pattern to mortality (Figure 5C).

Notably, for mortality, in low SDI countries, Somalia close to the frontier line; in middle SDI regions, Papua New Guinea, Solomon Islands, and other nations were near the frontier, while Afghanistan, Burkina Faso, and others were distant, with Afghanistan being the furthest from the frontier (Figure 5B). In high SDI regions, Singapore, Austria, and similar countries stayed close to the frontier, whereas Saudi Arabia diverged significantly. For DALYs, the relationship between national-level burdens and the frontier line was almost identical to that observed for mortality (Figure 5D).

### 3.4 Results of the predictive analysis for lead-attributable hypertensive CKD

**Figure 6:**
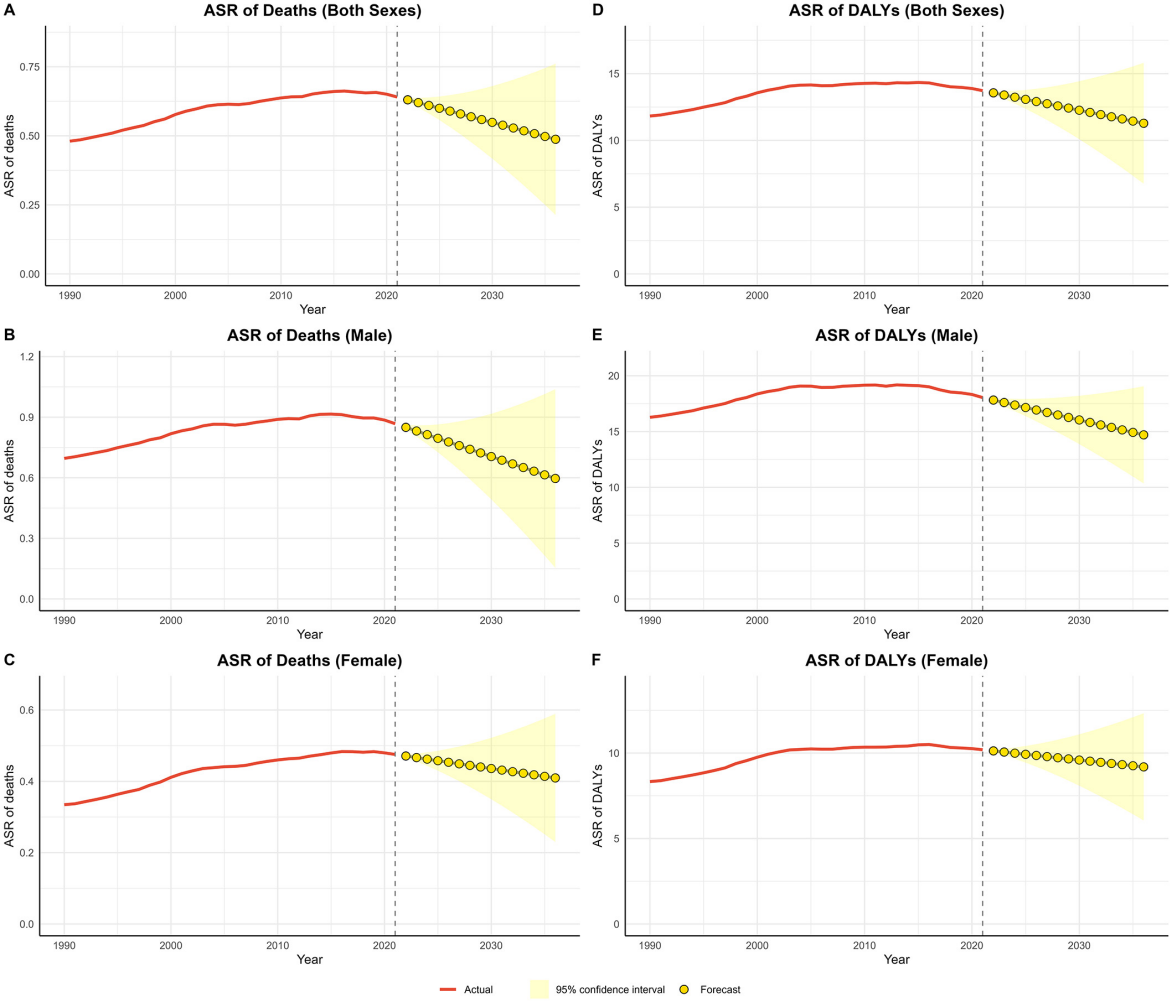
Predicted trends of lead-attributable hypertensive CKD over the next 15 years (2022–2036). **(A)** ASR of deaths of both; **(B)** ASR of deaths of male; **(C)** ASR of deaths of female; **(D)** ASR of DALYs of both; **(E)** ASR of DALYs of male; **(F)** ASR of DALYs of female. Red lines represent the true trend during 1990–2021; yellow dot lines and shaded regions represent the predicted trend and its 95% CI.

The forecast analysis of ASR for lead-attributable hypertensive CKD from 2021 to 2036 is shown in Figure 6. Validation of the ARIMA model demonstrated superior predictive performance in the female subgroup and the poorest performance in the male subgroup. Nevertheless, residual diagnostics confirmed homoscedasticity across all fitted models (Supplementary Table S3). For the age-standardized mortality rate (ASMR), the both sex trend has exhibited a decline since 2019, leading to a projection of continued decline over the next 15 years. Similarly, the ASDR has shown a downward trend since 2015, with the decline expected to persist through 2036. Subgroup analyses by sex revealed that both male and female trends were consistent with the overall pattern.

## 4 Discussion

### 4.1 Discussion on global and regional trend analysis results

From 1990 to 2021, there was a remarkable upward trend in global deaths and DALYs due to lead-attributable hypertensive CKD, with increases of 212.07% and 150.98%, respectively. The ASR also rose, highlighting the growing burden of this disease on global public health.

In 1990, high SDI regions had low numbers of death and DALYs cases and a low ASR. In contrast, low-middle SDI regions had the highest number of death cases, and low SDI regions had the highest ASR. By 2021, high-middle SDI regions had the lowest death cases, high SDI regions had the lowest ASR, middle SDI regions had the most death cases, and low SDI regions still had the highest ASR. This shows the complex relationship between economic development and disease burden. Low SDI regions are more vulnerable to lead pollution due to limited medical resources and inadequate public health measures (25, 26). High SDI regions have made progress in controlling lead exposure (27, 28). But middle SDI regions, which are still developing, may struggle with industrial restructuring and environmental pollution control, leading to a heavier disease burden (29).

Among the 21 GBD regions, in 2021, South Asia had the highest number of death cases, North Africa and the Middle East had the highest ASR, while Oceania had the lowest death and DALYs case, Eastern Europe had the lowest death and DALYs ASR. This shows that regional differences in environment, economy, and medical care affect disease distribution. For example, North Africa and the Middle East and South Asia could be affected by traditional fuel use and lax environmental regulation (30), while Eastern Europe and Oceania's well-developed environmental and medical systems have effectively controlled the disease (31).

Regarding relative changes, high SDI regions had the largest increases in death and DALYs cases and ASR, while low SDI regions had the smallest changes. This might mean high SDI regions have better disease monitoring and data collection, revealing potential cases (32). Or they may face new environmental and health challenges during development (29). Low SDI regions may have severe but underestimated disease burdens due to poor data (33).

Further EAPC analysis showed high SDI regions grew fastest, and some areas like Eastern Sub-Saharan Africa had negative DALYs growth. This could be due to regional differences in economic development (34), health investment, and policy implementation (35). High SDI regions have invested more in lead pollution control and improved medical care, so disease growth may be due to better monitoring revealing hidden cases (36, 37). Low SDI regions, with limited resources, have restricted disease growth but still face potential threats (37).

In terms of gender, men had higher numbers of death and DALYs cases and ASRs than female in both 1990 and 2021. But female had higher growth in ASR and magnitudes of change. This may be related to men's higher-risk occupational exposure (e.g., lead miners, construction workers, and mechanical workers) (38) and different lifestyle choices (e.g., smoking and alcohol consumption) (39). Female might respond differently to disease awareness and healthcare utilization, and more research is needed to understand these differences (40).

In summary, the global burden of CKD due to hypertension from lead exposure is complex and influenced by various factors. There are significant differences across regions and genders. Future strategies should be tailored to these differences, focusing on environmental governance and medical infrastructure development in low SDI regions, and further research into the reasons behind gender differences to address this public health challenge.

### 4.2 Synthesis of gender and age trends

From 1990 to 2021, the absolute numbers of deaths and DALYs from CKD due to lead exposure have shown an annual increase globally and across all SDI regions, with a slow overall upward trend in the ASR. Notably, males have higher numbers of deaths and DALYs than females before the age of 85–89 and 90–94 years, respectively, after which females experience higher numbers. The ASR for both mortality and DALYs increases with age across all groups, with males consistently showing higher values than females. Similar trends are observed across the five SDI regions.

Several factors may contribute to these patterns. Changes in population structure, including global population growth and aging, have expanded the susceptible population. Male participation in high-lead-exposure occupations such as mining and e-waste dismantling has decreased (41), while females are more exposed to lead pollution in household environments, such as from traditional cosmetics like “kajal” in South Asia, which contains lead levels exceeding standards by 20–50 times, and often lack protective measures (42). Gender imbalances in healthcare access have also increased the burden on females, with hypertension consultation rates for females being lower than those for males (43), leading to delays in screening and intervention for lead-related kidney damage.

Hormonal and metabolic differences affect gender-specific risks at different life stages. In early and middle adulthood, males are more prone to kidney damage due to androgen—promoted renal fibrosis, higher smoking rates, and occupational exposure (21, 44). In contrast, post-menopausal females experience accelerated vascular hardening due to estrogen deficiency, compounded by a high burden of age-related comorbidities, such as a diabetes (21, 45). Additionally, the physiological vulnerability varies significantly across age groups. The decline in kidney reserve function in the older population makes them more sensitive to lead toxicity and hypertension—related damage, resulting in a continuous increase in ASR with advancing age (46).

These interwoven factors lead to gender, age, and regional differences in disease burden. To address these issues, it is necessary to implement gender-specific interventions, such as occupational protection for males and blood pressure management for females during menopause, as well as age-stratified prevention and control measures, such as blood lead screening for early and middle-aged individuals and comorbidity management for the older population. Furthermore, efforts should be made to strengthen environmental governance in low SDI regions, such as phasing out lead—containing paints, and increase investment in medical resources, including training community health workers.

### 4.3 Relationships between SDI and lead-attributable hypertensive CKD burden

Globally, the ASRs of mortality and DALYs for CKD due to lead exposure show a significant negative correlation with the SDI. The lowest SDI regions bear the highest burden, while the highest SDI regions have the lowest burden. However, there is a minor inflection point at an SDI of approximately 0.5. When the SDI is between 0.5 and 0.6, the disease burden increases with the SDI, but after the SDI reaches around 0.6, the burden starts to decrease again. This phenomenon is closely related to different stages of socio-economic development. In low SDI regions, the absence of environmental governance (such as unregulated lead mining and illegal e-waste dismantling) (47–49), and insufficient medical resources (50). In high SDI regions, measures such as phasing out leaded gasoline and improving the medical insurance system have reduced blood lead levels (51, 52), thus effectively reducing the disease burden. In middle-income regions with an SDI between 0.5 and 0.6 (such as emerging economies in Southeast Asia), the “pollution paradox” arises due to surging lead demand during accelerated industrialization and lagging environmental regulations (53). However, as economic development drives upgrades in environmental protection technologies and improves access to healthcare, the disease burden decreases again when the SDI exceeds 0.6.

Our study suggests that differentiated strategies should be developed for different SDI levels: low SDI regions should prioritize addressing survival-related pollution and strengthening basic healthcare; middle SDI regions need to guard against development-related pollution and establish occupational health monitoring; and high SDI regions should focus on health inequalities and community environmental improvement. These approaches can help solve the regional prevention and control challenges of lead exposure-related kidney disease.

### 4.4 Prevention and control strategies for lead-attributable hypertensive CKD under frontier analysis

Frontier analysis reveals a significant impact of the SDI on the ASR of lead-attributable hypertensive CKD. Low-middle SDI regions show increasing deviation from the frontier line (ideal minimum burden) over time, indicating inadequate implementation of global best practices in lead pollution control and management. In contrast, high-middle SDI regions display divergent trends. Countries like Singapore and Austria, positioned on the frontier line, maintain low disease burdens through sustained policy interventions (54). Conversely, countries like Saudi Arabia, deviating from the frontier, face increased burdens due to lagging environmental policies or metabolic disease prevalence (55). Specifically, Somalia, a low-SDI country, approaches the frontier line due to its nomadic economy and unexpected international organizational interventions (56). Afghanistan, however, shows the greatest deviation due to war, militarized lead mining, and a collapsed healthcare system (57). Among middle-SDI countries, Papua New Guinea moves closer to the frontier through regulated mining and international medical collaboration (58), while Burkina Faso significantly deviates due to unprotected artisanal mining and drug shortages (59). Within high-SDI regions, disparities relate to policy enforcement and health inequalities (e.g., lead exposure in Saudi Arabia's construction) (60).

These findings suggest that low-SDI regions need to prioritize integrated “environment-health-conflict” interventions. Middle-SDI regions should establish resilient policy frameworks, and high-SDI regions should focus on equitable, socially targeted prevention and control to reduce global regional disparities in disease burden. Countries at the same SDI level that are far from the frontier line should learn from those closer to it.

### 4.5 Predictive analysis of lead-attributable hypertensive CKD and discussion on prevention and control strategies

The forecast analysis of ASR for lead-attributable hypertensive CKD from 2021 to 2036 presents a promising outlook. The projected continuous decline in both ASMR and ASDR over the next 15 years is a positive indication of the potential reduction in the disease burden. This trend may be attributed to the increasing awareness of the health impacts of lead exposure, the implementation of stricter environmental regulations, and improvements in healthcare systems. The consistent patterns observed in both males and females suggest that the interventions and policies in place are effectively targeting the entire population. However, it is crucial to maintain these efforts and continue monitoring the trends to ensure sustained progress in reducing the burden of lead-attributable hypertensive CKD.

### 4.6 Limitations

It is crucial to acknowledge several limitations of this study. Firstly, it is critical to emphasize the uncertainties inherent in attributing the burden of hypertensive CKD specifically to lead exposure. The estimates presented in this study rely on statistical models integrated into the GBD 2021 framework, which use causal inference methods to quantify the attributable fraction of disease burden based on exposure-response relationships. These models depend on multiple assumptions, including linearity in dose-response associations, completeness of exposure data, and exclusion of unmeasured confounding factors, which may introduce biases into the attribution results. Unlike direct observational data (e.g., prospective cohort studies with individual-level lead biomarker and clinical outcomes), our findings represent statistical extrapolations rather than direct evidence of causation. Thus, the absolute numbers of deaths and DALYs attributed to lead exposure should be interpreted with caution, as residual uncertainty may exist in disentangling lead's specific contribution from other concurrent risk factors. Secondly, the GBD database encompasses data from 1990 to 2021, during which time diagnostic criteria for diseases have undergone revisions. Such inconsistencies in diagnostic standards across different time periods may compromise the validity of the results. Moreover, a comprehensive assessment of disease burden should incorporate economic, familial, and social dimensions. Future research endeavors should thus adopt a multi—dimensional analytical framework to yield more precise and comprehensive outcomes. Additionally, translating the research findings on the impact of lead exposure on human health into actionable strategies, such as formulating public policies and guiding clinical decision—making, is imperative for effectively reducing the associated disease burden (37). Furthermore, the data from the GBD 2021 database may be affected by inconsistent data collection across regions; specifically, in resource-limited areas, under-reporting, misclassification, or incomplete data could lead to inaccurate disease burden estimates (58). Finally, the speculations in the discussions of result analysis in this paper, such as the gender differences stemming from occupational exposure and lifestyle, still require further correlational studies to be verified.

For future studies, it is recommended to consider the dose—response curves of lead exposure and specific diseases. Point-to-point quantitative results would facilitate a more accurate assessment of the burden imposed by lead exposure. Therefore, it would be more scientifically sound to compare the attributable burden of lead exposure among different populations in various regions, taking into account the spatiotemporal variations in lead exposure sources and the susceptibility profiles of specific populations. The results of this study provide valuable epidemiological evidence to inform targeted interventions for countries worldwide, aiming to mitigate lead exposure and its associated disease burden (37).

## 5 Conclusion

This study analyzed the global burden of lead-attributable hypertensive CKD from 1990 to 2021 and projected trends for the next 15 years. Results showed significant increases in deaths and DALYs globally, with complex variations across different SDI regions, genders, and age groups. The burden was heaviest in low SDI regions and lighter in high SDI regions, but with a minor inflection point at SDI 0.5. Frontier analysis revealed disparities in disease burden across countries, emphasizing the need for targeted interventions. Predictive analysis suggested a potential reduction in disease burden over the next 15 years. The study highlights the importance of addressing lead exposure to mitigate the burden of CKD due to hypertension and provides evidence for developing public health strategies. Future research should focus on refining dose-response relationships between lead exposure and CKD progression, exploring spatiotemporal variations in lead sources, and evaluating the cost-effectiveness of interventions tailored to SDI levels. By translating these findings into policy—such as updating global lead exposure standards and integrating CKD prevention into environmental health frameworks—this study can guide equitable reductions in the global burden of lead-attributable hypertensive CKD, ultimately improving renal health worldwide.

## Data Availability

Publicly available datasets were analyzed in this study. This data can be found here: https://vizhub.healthdata.org/gbd-results/.
