# Supplementary material for: Impact of lead exposure on global chronic kidney disease attributable to hypertension: deaths and disability-adjusted life years from 1990 to 2021 and projected trends for 2022–2036
Source: Front Public Health. 2025 Sep 23;13:1635877. doi: 10.3389/fpubh.2025.1635877 (PMC12500707; doi:10.3389/fpubh.2025.1635877)
Supplement: Supplementary file 1 [file Table_1.docx]

**Table S1** The case number and ASR of deaths of lead-attributable hypertensive CKD in 1990 and 2021, and its temporal trends from 1990 to 2021, categorized by global, SDI and 21 regions.

| **Location name** | **1990** |  | **2021** |  | **1990-2021** |  |  |
| --- | --- | --- | --- | --- | --- | --- | --- |
|  | **Case number (95% UI)** | **ASR (95% UI)** | **Case number (95% UI)** | **ASR (95% UI)** | **EAPC**  **(95% CI)** | **RC of numbers (%)** | **RC of ASR (%)** |
| Global | 16932(95% UI：-2255-37898) | 0.481(-0.063-1.083) | 52839(95% UI：-6416-117964) | 0.641(-0.077-1.435) | 1.05(0.91,1.19) | 212.07 | 33.25 |
| **Sex** |  |  |  |  |  |  |  |
| Male | 10309(95% UI：-1336-23293) | 0.696(-0.087-1.553) | 30595(95% UI：-3696-68177) | 0.868(-0.103-1.962) | 0.84(0.70,0.98) | 196.78 | 24.68 |
| Female | 6623(95% UI：-867-14952) | 0.334(-0.043-0.753) | 22244(95% UI：-2726-49759) | 0.475(-0.058-1.062) | 1.25(1.10,1.40) | 235.87 | 42.14 |
| **SDI** |  |  |  |  |  |  |  |
| High SDI | 1873(95% UI：-230-4207) | 0.172(-0.021-0.384) | 6882(95% UI：-852-15354) | 0.271(-0.034-0.604) | 1.97(1.77,2.17) | 267.36 | 57.11 |
| High-middle SDI | 2168(95% UI：-277-5005) | 0.261(-0.033-0.596) | 6517(95% UI：-730-14583) | 0.342(-0.038-0.760) | 1.04(0.88,1.20) | 200.67 | 31.18 |
| Middle SDI | 5555(95% UI：-722-12485) | 0.691(-0.086-1.576) | 19110(95% UI：-2346-43557) | 0.822(-0.099-1.872) | 0.70(0.48,0.92) | 243.99 | 19.01 |
| Low-middle SDI | 4680(95% UI：-637-10349) | 0.918(-0.121-1.976) | 14251(95% UI：-1748-31718) | 1.183(-0.142-2.623) | 0.80(0.68,0.93) | 204.48 | 28.89 |
| Low SDI | 2639(95% UI：-359-6184) | 1.486(-0.196-3.369) | 6030(95% UI：-780-13612) | 1.622(-0.201-3.653) | 0.28(0.17,0.39) | 128.5 | 9.2 |
| **Regions** |  |  |  |  |  |  |  |
| Andean Latin America | 207(95% UI：-25-480) | 1.117(-0.136-2.592) | 947(95% UI：-107-2102) | 1.683(-0.189-3.737) | 1.31(0.87,1.75) | 357.91 | 50.7 |
| Australasia | 42(95% UI：-5-90) | 0.198(-0.024-0.429) | 191(95% UI：-20-424) | 0.290(-0.031-0.644) | 1.83(1.41,2.26) | 356.3 | 46.65 |
| Caribbean | 236(95% UI：-30-525) | 0.981(-0.125-2.109) | 711(95% UI：-87-1562) | 1.301(-0.159-2.867) | 1.25(1.11,1.39) | 200.86 | 32.66 |
| Central Asia | 7(95% UI：-1-15) | 0.016(-0.002-0.034) | 33(95% UI：-4-73) | 0.048(-0.006-0.106) | 3.40(2.81,4.00) | 370.06 | 209.92 |
| Central Europe | 165(95% UI：-21-364) | 0.123(-0.015-0.272) | 326(95% UI：-39-706) | 0.135(-0.016-0.292) | 1.25(0.83,1.67) | 97.89 | 9.96 |
| Central Latin America | 921(95% UI：-111-2053) | 1.334(-0.157-2.960) | 4297(95% UI：-537-9504) | 1.803(-0.223-3.983) | 1.41(0.85,1.98) | 366.3 | 35.2 |
| Central Sub-Saharan Africa | 253(95% UI：-35-570 | 1.527(-0.197-3.425) | 702(95% UI：-90-1622) | 1.866(-0.230-4.325) | 0.59(0.53,0.64) | 177.88 | 22.2 |
| East Asia | 2979(95% UI：-352-7518) | 0.456(-0.050-1.097) | 8404(95% UI：-991-19370) | 0.440(-0.050-1.006) | -0.09(-0.23,0.05) | 182.14 | -3.63 |
| Eastern Europe | 46(95% UI：-6-99) | 0.017(-0.002-0.037) | 123(95% UI：-16-272) | 0.034(-0.004-0.077) | 2.15(1.57,2.73) | 168.18 | 99.68 |
| Eastern Sub-Saharan Africa | 1081(95% UI：-139-2535) | 1.893(-0.244-4.361) | 1890(95% UI：-227-4204) | 1.641(-0.190-3.663) | -0.72(-0.82,-0.63) | 74.83 | -13.32 |
| High-income Asia Pacific | 264(95% UI：-31-563) | 0.151(-0.017-0.325) | 906(95% UI：-102-2016) | 0.131(-0.015-0.291) | -0.09(-0.40,0.23) | 242.92 | -13.11 |
| High-income North America | 828(95% UI：-100-1887) | 0.225(-0.027-0.510) | 3392(95% UI：-437-7689) | 0.461(-0.060-1.048) | 2.81(2.57,3.06) | 309.58 | 104.64 |
| North Africa and Middle East | 2317(95% UI：-318-5246) | 1.738(-0.240-4.063) | 7556(95% UI：-930-16822) | 2.134(-0.257-4.812) | 0.83(0.76,0.90) | 226.04 | 22.73 |
| Oceania | 1(95% UI：-0-38) | 0.077(-0.008-0.189) | 5(95% UI：-1-12) | 0.095(-0.011-0.233) | 0.55(0.39,0.71) | 228.75 | 24.2 |
| South Asia | 3103(95% UI：-402-7096) | 0.638(-0.080-1.403) | 9166(95% UI：-1123-20533) | 0.743(-0.088-1.621) | 0.36(0.20,0.51) | 195.43 | 16.51 |
| Southeast Asia | 1646(95% UI：-229-3851) | 0.734(-0.100-1.704) | 5517(95% UI：-721-12490) | 0.981(-0.126-2.210) | 0.95(0.76,1.14) | 235.11 | 33.77 |
| Southern Latin America | 177(95% UI：-22-411) | 0.410(-0.049-0.951) | 438(95% UI：-51-1007) | 0.476(-0.056-1.094) | 0.66(0.28,1.04) | 147.9 | 16.24 |
| Southern Sub-Saharan Africa | 153(95% UI：-21-343) | 0.653(-0.089-1.476) | 589(95% UI：-76-1317) | 1.277(-0.159-2.895) | 1.90(1.31,2.49) | 285.21 | 95.48 |
| Tropical Latin America | 668(95% UI：-85-1464) | 0.861(-0.107-1.922) | 2165(95% UI：-267-4800) | 0.882(-0.109-1.948) | 0.20(-0.01,0.40) | 224.02 | 2.43 |
| Western Europe | 815(95% UI：-95-1780) | 0.138(-0.016-0.305) | 2736(95% UI：-303-6127) | 0.206(-0.023-0.459) | 1.86(1.70,2.02) | 235.95 | 49.7 |
| Western Sub-Saharan Africa | 1023(95% UI：-133-2383) | 1.437(-0.183-3.279) | 2747(95% UI：-376-6296) | 1.911(-0.245-4.309) | 0.84(0.68,1.01) | 168.45 | 33.01 |

Abbreviations: ASR, age-standardized rate; RC, relative change; EAPC, estimated annual percentage change; SDI, sociodemographic index; UI, uncertainty interval; CI, conﬁdence interval.

**Table S2** The case number and ASR of DALYs of lead-attributable hypertensive CKD in 1990 and 2021, and its temporal trends from 1990 to 2021, categorized by global, SDI and 21 regions.

| **Location name** | **1990** |  | **2021** |  | **1990-2021** |  |  |
| --- | --- | --- | --- | --- | --- | --- | --- |
|  | **Case number (95% UI)** | **ASR (95% UI)** | **Case number (95% UI)** | **ASR (95% UI)** | **EAPC**  **(95% CI)** | **RC of numbers (%)** | **RC of ASR (%)** |
| Global | 465569.641(-63870.725-1042328.853) | 11.825(-1.614-26.495) | 1168484.598(-148325.802-2520272.116) | 13.725(-1.735-29.739) | 0.55(0.41,0.70) | 150.98 | 16.06 |
| **Sex** |  |  |  |  |  |  |  |
| Male | 289816.603(-38807.928-650004.562) | 16.278(-2.151-36.051) | 698545.416(-87968.465-1512748.725) | 18.046(-2.250-39.507) | 0.42(0.27,0.56) | 141.03 | 10.86 |
| Female | 175753.039(-23697.714-398998.433) | 8.329(-1.120-18.821) | 469939.182(-60206.287-1046663.717) | 10.192(-1.305-22.710) | 0.71(0.56,0.86) | 167.39 | 22.36 |
| **SDI** |  |  |  |  |  |  |  |
| High SDI | 41375.457(-5380.451-90805.341) | 3.802(-0.497-8.343) | 114399.655(-14670.078-253014.204) | 5.101(-0.656-11.230) | 1.35(1.19,1.52) | 176.49 | 34.18 |
| High-middle SDI | 57404.537(-7761.070-132332.399) | 6.084(-0.811-13.831) | 128070.095(-15597.708-288960.447) | 6.574(-0.797-14.764) | 0.38(0.20,0.56) | 123.1 | 8.06 |
| Middle SDI | 155579.122(-20867.457-347389.218) | 15.572(-2.055-34.711) | 422324.651(-54124.396-915530.697) | 16.471(-2.087-35.920) | 0.29(0.07,0.51) | 171.45 | 5.77 |
| Low-middle SDI | 139322.568(-19503.128-312676.171) | 22.794(-3.167-50.632) | 354749.458(-44706.564-772721.798) | 25.789(-3.235-55.998) | 0.37(0.24,0.49) | 154.62 | 13.14 |
| Low SDI | 71466.830(-9813.935-163460.967) | 32.839(-4.509-74.479) | 147914.101(-19991.614-324239.771) | 32.009(-4.220-70.004) | -0.15(-0.22,-0.07) | 106.97 | -2.53 |
| **Regions** |  |  |  |  |  |  |  |
| Andean Latin America | 4908.300(-593.294-11207.450) | 23.924(-2.902-54.809) | 18322.007(-2179.245-41513.874) | 31.507(-3.757-71.002) | 0.84(0.43,1.24) | 273.29 | 31.69 |
| Australasia | 796.386(-104.700-1707.967) | 3.563(-0.462-7.709) | 2658.272(-312.419-5882.634) | 4.327(-0.520-9.510) | 1.03(0.68,1.37) | 233.79 | 21.46 |
| Caribbean | 6215.656(-812.278-13373.412) | 23.787(-3.118-50.928) | 15278.906(-1905.452-33101.945) | 28.377(-3.537-61.519) | 0.88(0.75,1.00) | 145.81 | 19.3 |
| Central Asia | 892.725(-127.477-1964.396) | 1.891(-0.270-4.183) | 1960.214(-273.457-4271.145) | 2.552(-0.356-5.541) | 0.90(0.60,1.20) | 119.58 | 34.99 |
| Central Europe | 4556.474(-618.976-9508.491) | 3.215(-0.428-6.792) | 6704.576(-877.720-14483.987) | 2.948(-0.386-6.282) | 0.41(0.11,0.71) | 47.14 | -8.3 |
| Central Latin America | 22674.723(-2879.501-48311.852) | 28.193(-3.553-61.555) | 91912.910(-12220.106-200164.221) | 37.077(-4.899-81.507) | 1.24(0.64,1.84) | 305.35 | 31.51 |
| Central Sub-Saharan Africa | 7123.857(-1024.266-16124.331) | 32.804(-4.524-72.406) | 18068.197(-2341.265-41652.241) | 36.271(-4.652-82.519) | 0.26(0.20,0.33) | 153.63 | 10.57 |
| East Asia | 86060.652(-10399.173-212351.177) | 10.532(-1.264-25.691) | 181785.065(-22852.305-426021.790) | 8.708(-1.081-20.338) | -0.53(-0.70,-0.36) | 111.23 | -17.32 |
| Eastern Europe | 2621.728(-373.834-5663.791) | 1.005(-0.143-2.204) | 4067.287(-582.187-8677.037) | 1.186(-0.168-2.529) | 0.23(0.05,0.41) | 55.14 | 18.08 |
| Eastern Sub-Saharan Africa | 26206.048(-3416.123-61614.623) | 37.955(-4.906-88.473) | 40501.669(-4986.680-88585.982) | 28.459(-3.454-63.026) | -1.23(-1.33,-1.12) | 54.55 | -25.02 |
| High-income Asia Pacific | 6062.772(-770.521-13010.480) | 3.160(-0.398-6.667) | 13582.554(-1670.309-29836.493) | 2.379(-0.303-5.198) | -0.54(-0.78,-0.29) | 124.03 | -24.71 |
| High-income North America | 16832.628(-2126.787-37351.611) | 4.732(-0.601-10.457) | 55170.114(-7281.871-124218.331) | 8.024(-1.064-18.097) | 2.13(1.89,2.36) | 227.76 | 69.57 |
| North Africa and Middle East | 56243.792(-7586.810-126498.344) | 35.231(-4.810-78.669) | 157684.934(-19930.769-353917.128) | 38.352(-4.794-85.833) | 0.41(0.36,0.46) | 180.36 | 8.86 |
| Oceania | 50.231(-6.128-124.491) | 1.998(-0.237-4.843) | 150.800(-18.775-375.239) | 2.340(-0.287-5.686) | 0.42(0.26,0.59) | 200.21 | 17.12 |
| South Asia | 106389.016(-14289.068-235047.942) | 18.015(-2.399-39.777) | 258616.460(-33125.696-567284.520) | 18.185(-2.305-39.475) | -0.08(-0.18,0.02) | 143.09 | 0.95 |
| Southeast Asia | 47825.342(-6688.588-112181.729) | 17.971(-2.511-42.077) | 133670.471(-17815.038-306216.836) | 20.890(-2.757-47.354) | 0.51(0.34,0.68) | 179.5 | 16.24 |
| Southern Latin America | 3906.603(-507.873-9084.917) | 8.596(-1.115-19.909) | 7494.857(-935.139-16970.013) | 8.396(-1.052-18.922) | 0.07(-0.28,0.42) | 91.85 | -2.33 |
| Southern Sub-Saharan Africa | 4083.423(-565.921-9261.954) | 14.800(-2.023-33.507) | 13836.699(-1867.563-30517.460) | 25.176(-3.343-55.061) | 1.47(0.89,2.04) | 238.85 | 70.11 |
| Tropical Latin America | 18802.518(-2444.405-39664.545) | 20.381(-2.634-43.516) | 44342.202(-5795.332-96347.643) | 17.508(-2.279-38.169) | -0.50(-0.72,-0.27) | 135.83 | -14.1 |
| Western Europe | 17625.626(-2286.104-37458.953) | 3.067(-0.396-6.522) | 39102.850(-4705.319-85999.803) | 3.366(-0.413-7.290) | 0.65(0.53,0.77) | 121.85 | 9.73 |
| Western Sub-Saharan Africa | 25691.141(-3369.921-59471.224) | 29.997(-3.949-69.540) | 63573.553(-9134.110-142477.388) | 35.350(-4.930-80.129) | 0.43(0.25,0.61) | 147.45 | 17.85 |

Abbreviations: ASR, age-standardized rate; RC, relative change; EAPC, estimated annual percentage change; SDI, sociodemographic index; UI, uncertainty interval; CI, conﬁdence interval.

**Table S3** Validation of ARIMA's suitability for predictions

| Measure | Sex | AIC | BIC | LB p value | RMSE | MAE | MAPE |
| --- | --- | --- | --- | --- | --- | --- | --- |
| Deaths | Both | -244.6899915 | -243.2887941 | 0.261241437 | 0.035060652 | 0.028615798 | 4.399513139 |
| Deaths | Male | -215.9611927 | -214.5599953 | 0.148515751 | 0.061997849 | 0.052678197 | 5.938272259 |
| Deaths | Female | -270.0079441 | -268.6067467 | 0.266975966 | 0.018405145 | 0.013918 | 2.904418219 |
| DALYs | Both | -76.39817412 | -74.99697674 | 0.308450346 | 0.361422251 | 0.290265423 | 2.088023208 |
| DALYs | Male | -51.80353667 | -49.00114191 | 0.527223651 | 0.630143135 | 0.52734216 | 2.870178798 |
| DALYs | Female | -98.45314942 | -97.05195204 | 0.775896388 | 0.178946589 | 0.149432411 | 1.452982527 |

Abbreviations: AIC: Akaike Information Criterion, BIC: Bayesian Information Criterion, LB p value: Ljung-Box Test p-value, RMSE: Root Mean Square Error, MAE: Mean Absolute Error, MAPE: Mean Absolute Percentage Error.
